# Modeling and analysis of vehicle path dispersion at signalized intersections using explainable backpropagation neural networks

**DOI:** 10.1016/j.fmre.2023.08.008

**Published:** 2023-11-05

**Authors:** Jing Zhao, Ruoming Ma, Jian Sun, Rongji Zhang, Cheng Zhang

**Affiliations:** a Department of Traffic Engineering, University of Shanghai for Science and Technology, Shanghai 200093, China; b College of Transportation Engineering, Tongji University, Shanghai 201804, China

**Keywords:** Path dispersion, Driving behaviors, Signalized intersections, Backpropagation neural network, Operation order, Empirical analysis

## Abstract

The dispersion of vehicular paths is a common phenomenon in the inner area of signalized intersections due to heterogeneous driver behavior and interactions. This study aims to develop an explainable neural network-based model to describe the vehicle path dispersion by exploring the relationship between the path dispersion and external factors. A backpropagation neural network model was established to analyze the effects of external factors on the dispersion of through and left-turn paths based on real trajectory data collected from 20 intersections in Shanghai, China. Twelve influencing factors in varying geometric, traffic, signalization, and traffic management conditions were considered. The predictive power and transferability of the model were verified by applying the trained model on the four new intersections. The contributions of the influencing factors on the path dispersion were explored based on the neural interpretation diagram, relative importance of influencing factors, and sensitivity analysis to offer explanatory insights for the proposed model. The results show that the mean absolute percentage errors of the path dispersion models for the through and left-turn movements are only 14.67% and 17.65%, respectively. The through path dispersion is primarily influenced by the number of exit lanes, the offset degree between the approach and exit lanes, and the traffic saturation degree on the through lane. In contrast, the path dispersion of the left turn is mainly affected by the number of exit lanes, the left-turn angle, and the setting of guide lines.

## Graphical abstract

**Figure:**
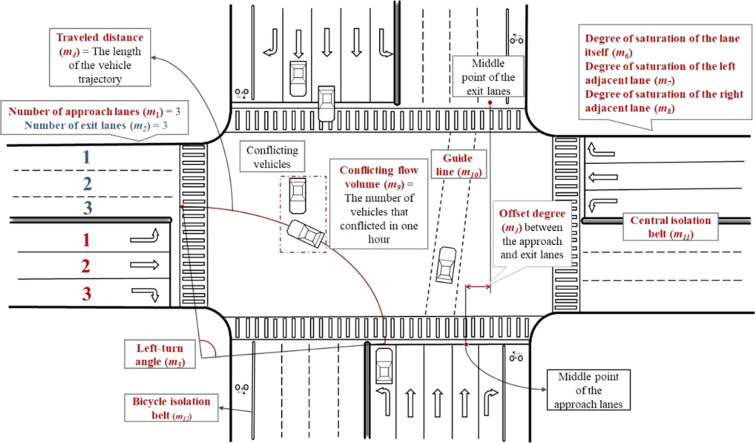

## 1 Introduction

The dispersion of vehicular paths is a common phenomenon in the inner area of signalized intersections. In the inner area of intersections, drivers can select numerous paths when they pass through the two-dimensional space of intersections from an approach lane to an exit lane. A lower consciousness of traffic lanes makes it difficult for vehicles to move along the expected traffic flow lines. This leads to the dispersion of vehicular paths, which further affects the running efficiency and conflicts inside intersections. In the existing literature [1], path dispersion, which reflects the distribution of vehicular path inside signalized intersections, was used to quantify this phenomenon. Studies have found that the dispersion of paths has an impact on the traffic efficiency of intersections [2,3]. Therefore, it is important to depict the path dispersion inside an intersection and determine its influencing factors to guide the design, control, and management of intersections and further improve the operation order. This paper sets its scope to the modeling of vehicle path dispersion. We first review the relevant literature.

### 1.1 Related work

The literature describing the operating state of signalized intersections can be categorized into two categories: (1) models of vehicular traffic flow of intersections; (2) models of overall performance of intersections based on empirical data.

Traffic flow models can describe the interaction between vehicles. The methods used to model the microscopic vehicular traffic flow at intersections mainly consist of (a) car-following and lane-changing models, (b) cellular automata (CA) models, (c) social force models, and (d) optimal control models.

Car following and lane changing are basic microscopic driving methods. They can be utilized to analyze the traffic features of two vehicles staying in the same lane or moving from one lane to another [4], [5], [6], [7], and they are widely used in commercial microscopic simulation software. Lots of studies indicated that the moving of vehicles is greatly affected by the driver behavior and performance, such as the fatigue driving [8], emission consideration [9], speed guidance [10,11], sight distance [12], braking process [13], interaction with pedestrians [14], driving habits of headways [15], emotional state [16], subjective awareness of speed information [17], and the mixed traffic of autonomous and human driven vehicles [18], [19], [20].

The CA model is another widely used model for analyzing the traffic efficiency and safety of intersections [21], [22], [23]. Spyropoulou [24] simulated the CA model at signalized intersections and analyzed the influence of dynamic random parameters on the saturation flow rate. Chai et al. [25,26] described the traffic conflict between left-turn and through vehicles at signalized intersections using the CA model and discussed the impact of traffic flow and left-turn phase on conflicting vehicles. The CA model can also be used to simulate mixed traffic flow at intersections. Vasic and Ruskin [27] studied the conflicts between vehicles and bicycles. Tang et al. [28] described the phenomenon of random lane-changing and retrograde behaviors of electric bicycles near signalized intersections and examined the effect of electric bicycles on intersection congestion.

The social force model was originally proposed by Helbing et al. [29] to obtain the feature of microscopic pedestrian dynamics. It quantifies the interactions between pedestrians and the external space and considers them as gravitational and repulsive forces, respectively. This model was further improved to describe the movement of vehicles. A three-layered “plan–decision–action” framework was proposed by Ma et al. [30] to solve the limitation of turning vehicles in traditional one-dimensional models.

The optimal control model is mainly used for vehicular trajectory planning in an automatic driving environment [31], [32], [33], [34]. Bichiou and Rakha [35] proposed an optimal control algorithm to plan the vehicular trajectory at intersections by taking both the speed change rate and vector direction as control variables. Zhao et al. [36] proposed a model for vehicle trajectory planning using distance as an independent variable that can decouple speed and path. Therefore, the model can represent the driving behavior of drivers, including turning the steering wheel and pushing the brake or throttle pedals. They further extended the model to describe human-driven vehicle maneuvers under interactions, which can endogenously give the order of vehicles in case of crossing paths [37].

In existing studies, external environment factors, such as road geometry and signal control, and vehicle interactions are considered in the traffic flow model. However, an overview of the path dispersion caused by these factors at intersections has never been provided.

To evaluate the overall operational performance of intersections, models were proposed from efficiency and safety aspects based on empirical data. For evaluating the operational efficiency at intersections, capacity, delay, and queue length are commonly used indicators. The highway capacity manual [38], a typical capacity calculation method for signalized intersections, obtains the capacity of lane groups at intersections by calculating the saturation flow rate and effective green split. Existing studies focus on exploring the influencing factors of the saturation flow rate under various geometric [39], [40], [41], [42], signalization [43], [44], [45], [46], and traffic conditions [47], [48], [49], [50]. The delay at signalized intersections refers to the time lost owing to traffic flow interruptions caused by signal control. Webster proposed a steady-state delay model for intersections according to the features of vehicle arrival [51]. Uniform delay and random delay can be calculated by inputting parameters such as vehicle arrival rate, saturation flow rate, and signal timing. Intersection delay is related to the features of vehicle arrival and departure when the arrival rate fluctuates significantly. Therefore, based on accurate trajectory data, comparing theoretical models with reality and constantly improving the accuracy have become hot topics in recent years [52,53]. The queue length of signalized intersections is an indicator of the congestion level at intersections in terms of space. Traditionally, it can be estimated based on the arrival–departure cumulative curve and traffic wave theory. Currently, studies on real-time dynamic estimation of queue length at intersections based on floating car trajectory data are also conducted [54].

For the evaluation of traffic safety at intersections, the common indices are mainly divided into two groups: direct indices based on accident data and surrogate safety measures (SSMs) based on traffic conflicts. The typical SSMs include time to collision (TTC), deceleration rate to avoid a crash (DRAC), and post-encroachment time (PET). The TTC was first proposed by Hayward [55]. It indicates the time for the vehicle behind to collide with the vehicle in front if both maintain present speed and trajectory and the speed of the rear vehicle is greater than that of the front vehicle. Ozbay et al. [56] considered the acceleration changes in conflicting vehicles and improved the TTC calculation model. The TTC calculation methods mentioned above are mainly applied to the car-following behavior. Considering the multilane condition, Wang and Stamatiadis [57] proposed a TTC calculation method that considers lane changing. Xing et al. [58] proposed a model to deal with the problem of mixed traffic flow upstream of the tollbooth. For the no-lane condition, Charly and Mathew [59] proposed an optimized calculation method that can predict whether two vehicles will have conflicts by analyzing their trajectory overlaps. The DRAC is a safety indicator for judging whether a collision will occur based on the vehicle's running feature, and it has the same assumption as the TTC [56]. It was originally used in the simulation software of the Federal Highway Administration. The DRAC represents the deceleration rate for braking to avoid a rear-end collision. If the rate is greater than the maximum available deceleration rate for the vehicle during braking, traffic conflicts will result [60]. The PET is the time difference between two vehicles passing through the same conflict area, as proposed by Hyden [61]. The conflict area here can be a plane, line, or point. Generally, this indicator is used for vehicles with crossing trajectories.

It can be thus concluded that although much is known about the interaction between vehicles, the overview path dispersion has not been specifically analyzed, and influencing factors have never been explored. It is interesting to know what external factors cause the overview path dispersion at intersections.

### 1.2 Objective and contribution

This study aims to explore the external influencing factors of overview vehicle path dispersion inside signalized intersections based on empirical data. The real trajectory data under various external factors of geometric, traffic, signal control, and traffic management conditions were collected from 20 intersections in Shanghai, China. The entire dataset was divided into three subsets for training, validation, and testing, respectively. The predictive power and transferability of the model were verified by applying the trained model to the four new intersections. The explanatory insights for the proposed model were offered by exploring the contributions of the influencing factors on the path dispersion based on the neural interpretation diagram, relative importance of influencing factors, and sensitivity analysis. The results can explore the primary influencing factors of the path dispersion phenomenon, which can reflect the operation order of intersections.

The remainder of this paper is organized as follows. Section 2 presents the raw data analysis, including data collection and pre-processing. Section 3 describes the development of the backpropagation neural network (BPNN) model for predicting path dispersion at signalized intersections. Section 4 provides explanations of the model results and effects of various factors on path dispersion from multiple perspectives. Section 5 summarizes the research results and discusses future research directions.

## 2 Data collection overview

### 2.1 Field data collection

**Fig. 1:**
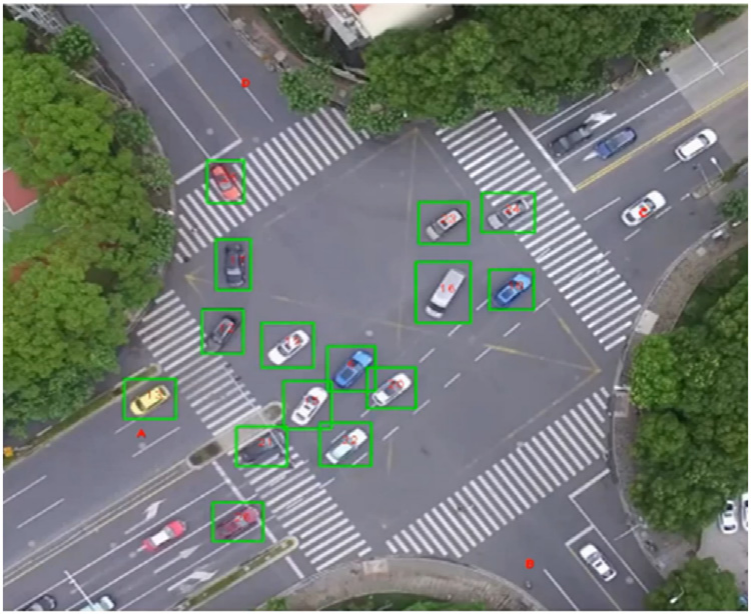
**User interface of the video recognition software**.

To investigate the influencing factors of path dispersion in signalized intersections, 20 signalized intersections in Shanghai, China, were selected. The selection of the studied intersections is based on their variations in geometric, traffic, signal control, and traffic management conditions, all of which were identified as potential influencing factors from the literature. For geometric conditions, we considered the number of approach lanes (NAL) and exit lanes (NEL) and the distance offset between the approach and exit lanes (DO). Regarding the signalization conditions, we considered the presence of the protected left-turn phases (LP). In terms of traffic management conditions, we considered the presence of guidelines (GL), central isolation belts (CID), and bicycle isolation belts (BIB). Table 1 presents the descriptive statistics of the surveyed intersections. Detailed information is shown in Appendix A. All data in this study were captured using an unmanned aerial vehicle during the morning peak (between 7:00 and 9:00) in September and October 2019, in good weather conditions. Then, using a specially developed video recognition software [36], we collected the position of each vehicle at each frame (1/24 s) to obtain the real vehicular trajectories, as shown in Fig. 1.

**Table 1:** **Descriptive statistics of surveyed intersections**.

Influencing factors	Groups	Percentages
Number of approach lanes (NAL)	1	6.25%
	2	41.25%
	3	36.25%
	4	11.25%
	5	3.75%

Number of exit lanes (NEL)	1	31.25%
	2	55.00%
	3	10.00%
	4	2.50%

Distance offset between the approach and exit lanes (DO)	1-Large	23.08%
	0-Small	76.92%

Presence of the protected left-turn phases (LP)	1-Yes	31.65%
	0-No	68.35%

Presence of guidelines (GL)	1-Yes	23.75%
	0-No	76.25%

Presence of central isolation belts (CID)	1-Yes	43.75%
	0-No	56.25%

Presence of bicycle isolation belts (BIB)	1-Yes	43.75%
	0-No	56.25%

### 2.2 Descriptive analysis

**Fig. 2:**
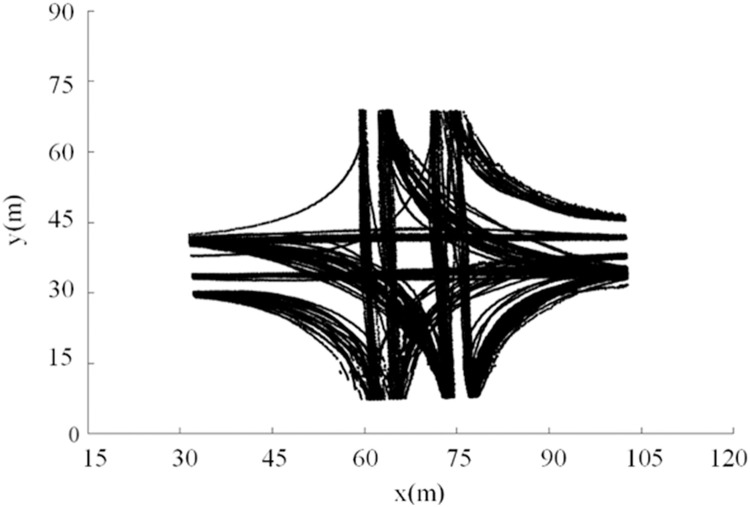
**Vehicular trajectories at Yangtai Road–Zhentai Road intersection**.

A total of 4394 vehicle trajectories were collected. Taking the intersection of Yangtai Road and Zhentai Road as an example, the vehicle trajectories are displayed in Fig. 2, which exhibits significant dispersion. The descriptive statistics of the maneuvers from the surveyed intersections are shown in Table 2. The description and analysis of the influencing factors of path dispersion are presented in the following section.

**Table 2:** **Descriptive statistics of movements**.

Movements	Parameters	Mean	Q1 (25%)	Q2 (50%)	Q3 (75%)	Standard deviation
Left turn	Speeds (m/s)	4.705	2.623	4.792	6.717	2.688
	Curvature (m^−1^)	0.033	0.026	0.03	0.037	0.011
	Longitudinal acceleration (m/s^2^)	0.199	−0.809	0.144	1.25	1.625
	Lateral acceleration (m/s^2^)	0.699	0.036	0.538	1.537	0.769
	Speeds (m/s)	8.89	6.65	9.064	11.159	3.471
	Curvature (m^−1^)	0.004	0.001	0.002	0.003	0.007
Through	Longitudinal acceleration (m/s^2^)	0.324	−0.738	0.316	1.395	1.717
	Lateral acceleration (m/s^2^)	0.017	−0.682	0.005	0.725	1.025
	Speeds (m/s)	4.601	2.811	4.513	6.159	2.495
	Curvature (m^−1^)	0.039	0.029	0.038	0.047	0.015
Right turn	Longitudinal acceleration (m/s^2^)	0.202	−0.88	0.183	1.311	1.697
	Lateral acceleration (m/s^2^)	−0.473	−1.194	−0.435	−0.019	0.957

## 3 Path dispersion prediction model development

Owing to the complex relationship between the path dispersion and various influencing factors at signalized intersections, an artificial neural network (ANN) was used to explore their connections. The BPNN is a type of ANN widely used in various research fields to conduct network self-learning through the method of propagating information forward and errors backward. The BPNN is usually composed of an input layer, a hidden layer, and an output layer. The hidden layer can be a single or multiple layers but usually, a single hidden layer can solve most of the problems. The model determines whether the neural network can achieve the training goal by adjusting the connection weights, transfer functions, and thresholds of each neuron in each layer.

### 3.1 Path dispersion and influencing factors

#### 3.1.1 Output variable: path dispersion

**Fig. 3:**
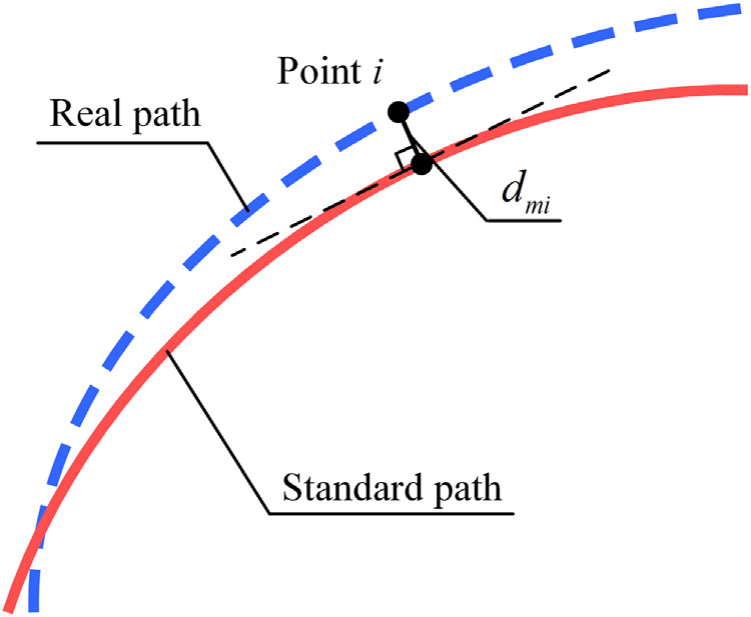
**Distance between point*****i*****in real and standard paths**.

The output variable in this study is the path dispersion of vehicles, which reflects the distribution of vehicular paths at signal control intersections. Generally, the standard deviation can be used to describe the degree of dispersion. Considering that the vehicular path is a two-dimensional curve, the path dispersion of a certain travel direction at an intersection can be defined as the standard deviation of the distance between each real path and the median path of all paths, as shown in Eq. 1. Fig. 3 presents the interpretation diagram for distance $d_{mi}$ between point *i* in a path and median path:

(1) $$\sigma_{m}^{P}=\sqrt{\frac{1}{n}\sum_{i=1}^{n}{d_{mi}}^{2}}$$

where $\sigma_{m}^{P}$ denotes the index of the path dispersion for travel direction *m*, $d_{mi}$ is the distance between point *i* and the median path of travel direction *m*, and *n* is number of points in the path of travel direction *m*.

#### 3.1.2 Input variables: potential influencing factors

**Fig. 4:**
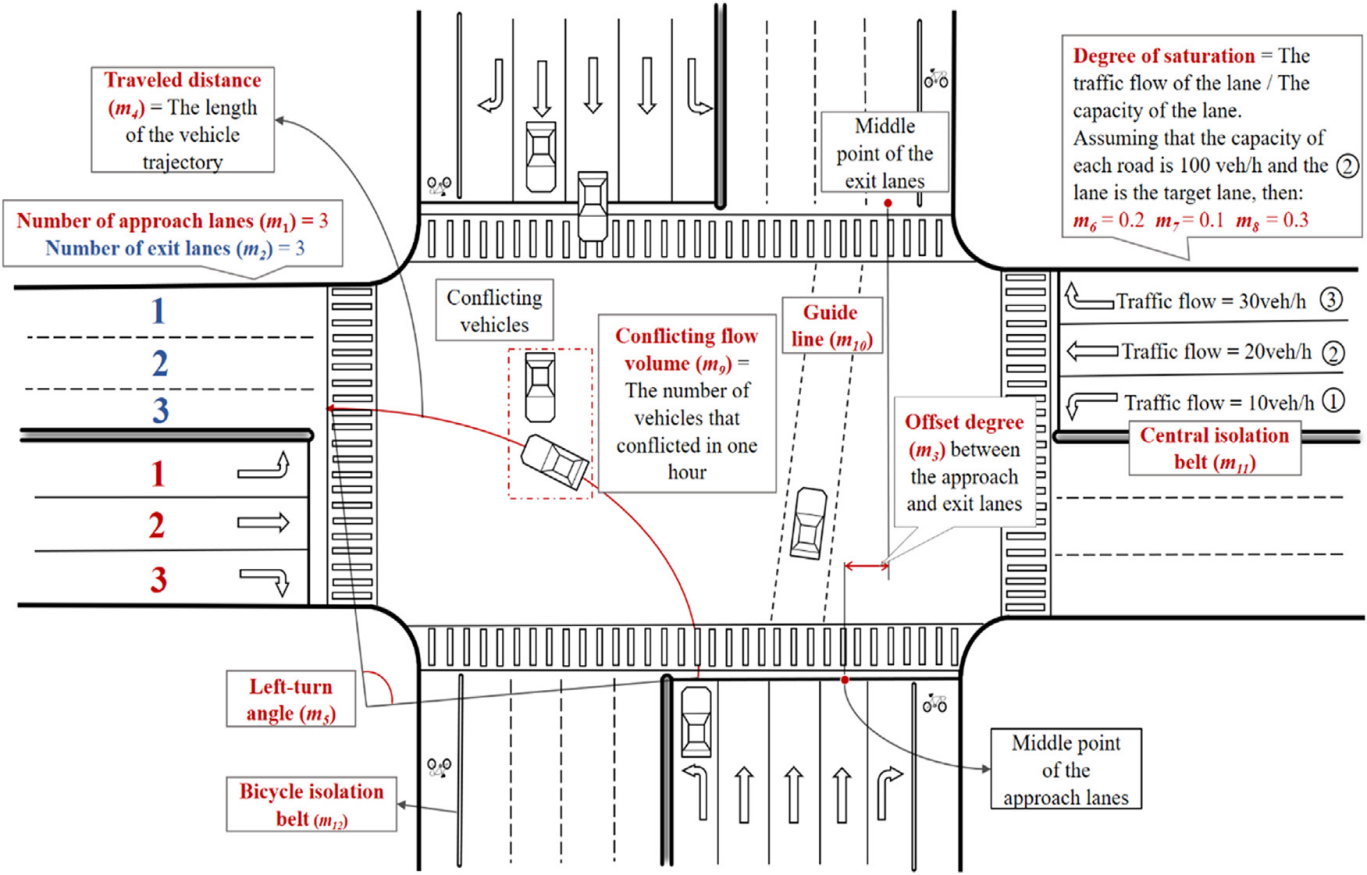
**Potential influencing factors**.

The input variables were selected from the following four aspects: geometric conditions, traffic conditions, signalization conditions, and traffic management conditions. The geometric conditions consist of the number of approach and exit lanes (*m*_1_ and *m*_2_), offset degree between the approach and exit lanes (*m*_3_), traveled distance (*m*_4_), and left-turn angle (*m*_5_). The traffic conditions include the degree of saturation of the lane itself (*m*_6_) and the degrees of saturation of the left and right adjacent lanes (*m*_7_ and *m*_8_). The signalization condition is composed of the conflicting flow volume (*m*_9_). It is equal to zero when the protected left-turn phase is adopted. The traffic management conditions consist of the setting of guidelines (*m*_10_), central isolation belts (*m*_11_), and bicycle isolation belts (*m*_12_). The description of the input variables is presented in Table 3. In summary, there are 12 potential influencing factors, as shown in Fig. 4. Note that the left-turn angle is only used for examining the path dispersion of the left turn, while the offset degree between the approach and exit lanes is only used for examining the path dispersion of the through movement. The other factors are considered for both the through and left-turn movements.

**Table 3:** **Description of input variables**.

Types	Variable	Description
Geometric conditions	*m*_1_: Number of approach lanes	Discrete variable
	*m*_2_: Number of exit lanes	Discrete variable
	*m*_3_: Offset degree between the approach and exit lanes	Continuous variable, which is measured by the distance of the vertical line from the middle point of the exit lane to the line of the approach lane, in m
	*m*_4_: Traveled distance	Continuous variable, in m
	*m*_5_: Left-turn angle	Continuous variable, in rad
Traffic conditions	*m*_6_: Degree of saturation of the lane itself	Continuous variable
	*m*_7_: Degree of saturation of the left adjacent lane	Continuous variable; *m_7_* = 0 when there is no left adjacent lane
	*m*_8_: Degree of saturation of the right adjacent lane	Continuous variable; *m*_8_ = 0 when there is no right adjacent lane
Signalization conditions	*m*_9_: Conflicting flow volume	Continuous variable, in veh/h; *m_9_* = 0 when the protected left-turn phase is adopted
Management conditions	*m*_10_: Setting of guideline	Binary variable; 1 = yes, 0 = no
	*m*_11_: Setting of central isolation belt	Binary variable; 1 = yes, 0 = no
	*m*_12_: Setting of bicycle isolation belt	Binary variable; 1 = yes, 0 = no

### 3.2 BPNN model establishment

**Fig. 5:**
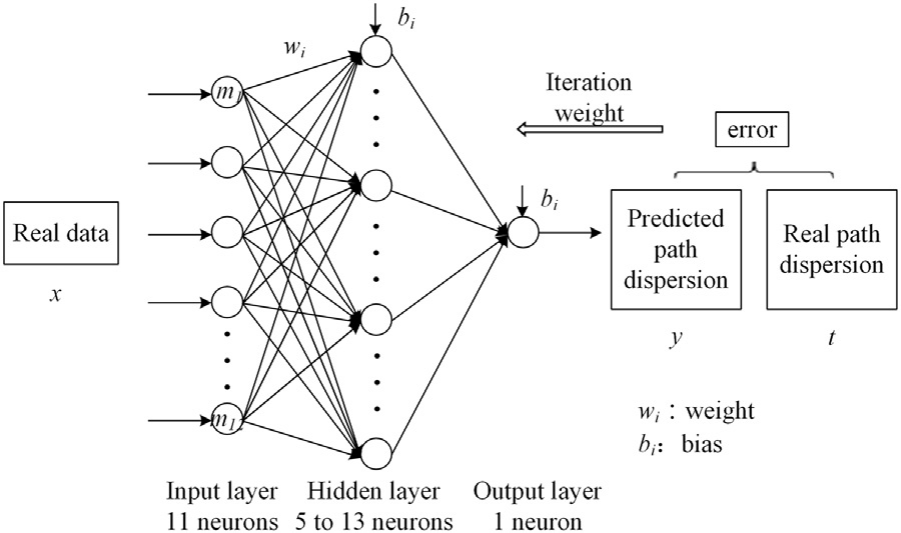
**Architecture of the BPNN model**.

The proposed BPNN has three layers. The numbers of neurons in the input and output layers are 11 and 1, respectively. To find a better architecture for the BPNN model, three types of neuron numbers in the hidden layer were tested: 5, 9, and 13. The architecture of the BPNN model used in this study is shown in Fig. 5. The principle of the model is given by [62]:

(2) $$y=f\left(\sum_{i=1}^{n}m_{i}w_{i}+b\right)$$

where $m_{i}$ denotes the neurons in the input layer, $w_{i}$ is the weight between neurons, b is the bias, and f and *y* are the activation function and neurons in the output layer, respectively.

The BPNN usually utilizes the mean square error *E*, given by Eq. 3, as an indicator for evaluating the training performance of the model. *E* can be minimized by gradually correcting the network weight, as shown in Eq. 4. The sigmoid activation function, tansig transfer function, and Levenberg–Marquardt training algorithm were used in this study. The paper utilizes the Levenberg-Marquardt algorithm as it is the integration of the steepest descent and Gauss-Newton methods, which makes it robust and efficient in solving nonlinear least-squares problems with noisy or ill-conditioned data [63,64]. The error *E* was calculated using the mean square error function. The number of training display intervals was 10. The learning rate, number of iterations, and model training error target were set as 0.1, 1000, and 0.01, respectively:

(3) $$E=\frac{1}{n}\sum_{i=1}^{n}w_{i}{\left(y_{i}-\bar{y_{i}}\right)}^{2}$$

(4) $$\min E\left(e^{T}e\right)=\min E\left[{\left(t_{i}-y_{i}\right)}^{T}\left(t_{i}-y_{i}\right)\right]$$

where *n* denotes the number of points (samples), *e* is the network error vector, $y_{i}$ is the output of the model, and $t_{i}$ is the expected output of the corresponding sample.

### 3.3 BPNN modeling results

The entire dataset was divided into three subsets: the data from intersections 1–16 were used for model training (80% of the data from intersections 1–16) and validation (20% of the data from intersections 1–16), whereas those of the remaining four intersections (intersections 17–20) were used to test the transferability of the established model. The determination of the fitting effect of the BPNN model was based on two indicators: the coefficient of determination (*R*^2^) and mean absolute error (MAE). In addition, we choose multiple linear regression as a baseline to further test the superiority of the model.

**Fig. 6:**
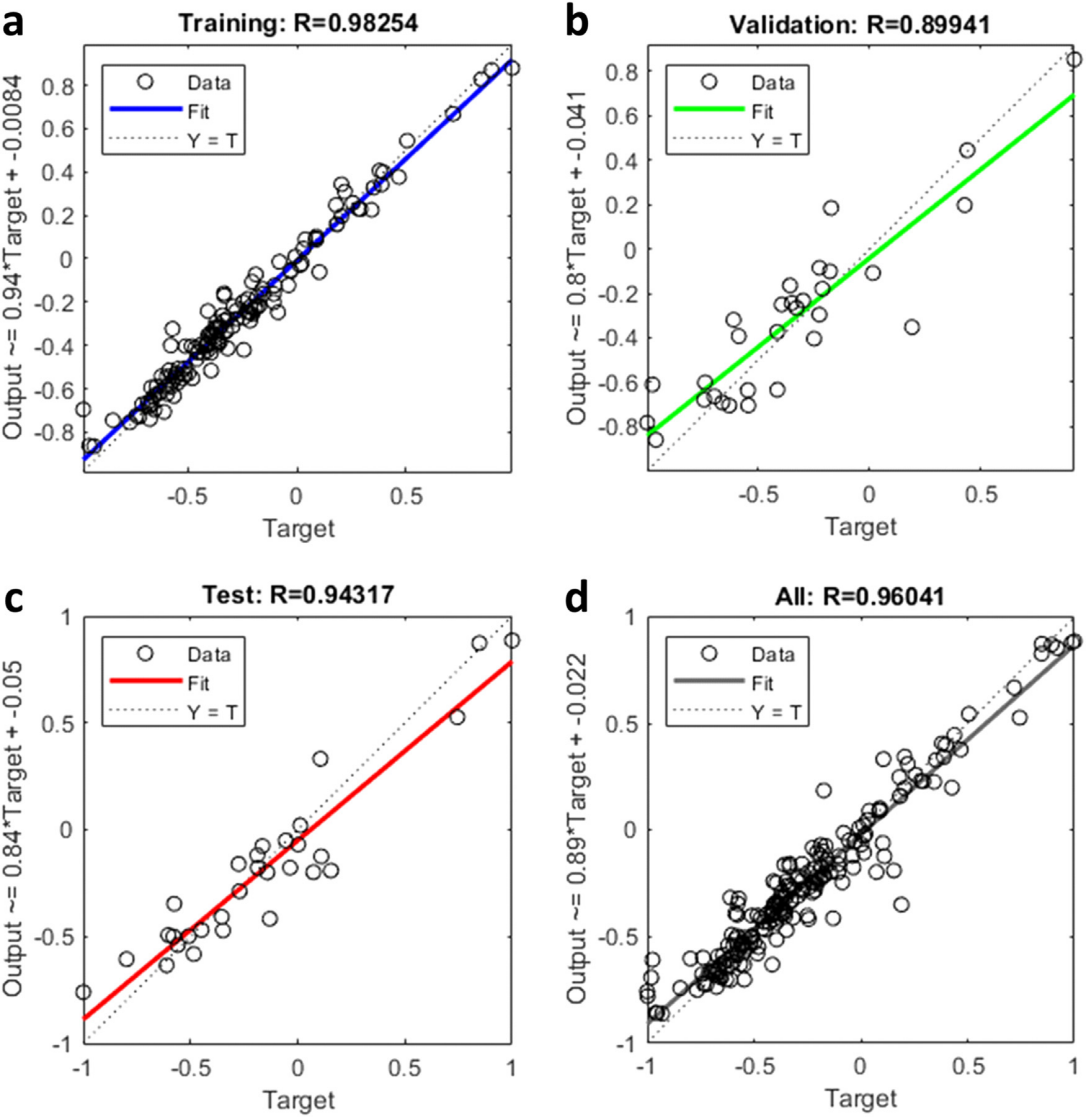
**BPNN model for the through movement (N13)**. (a) Performance of the training. (b) Performance of the validation. (c) Performance of the test. (d) Performance of the all data.

**Fig. 7:**
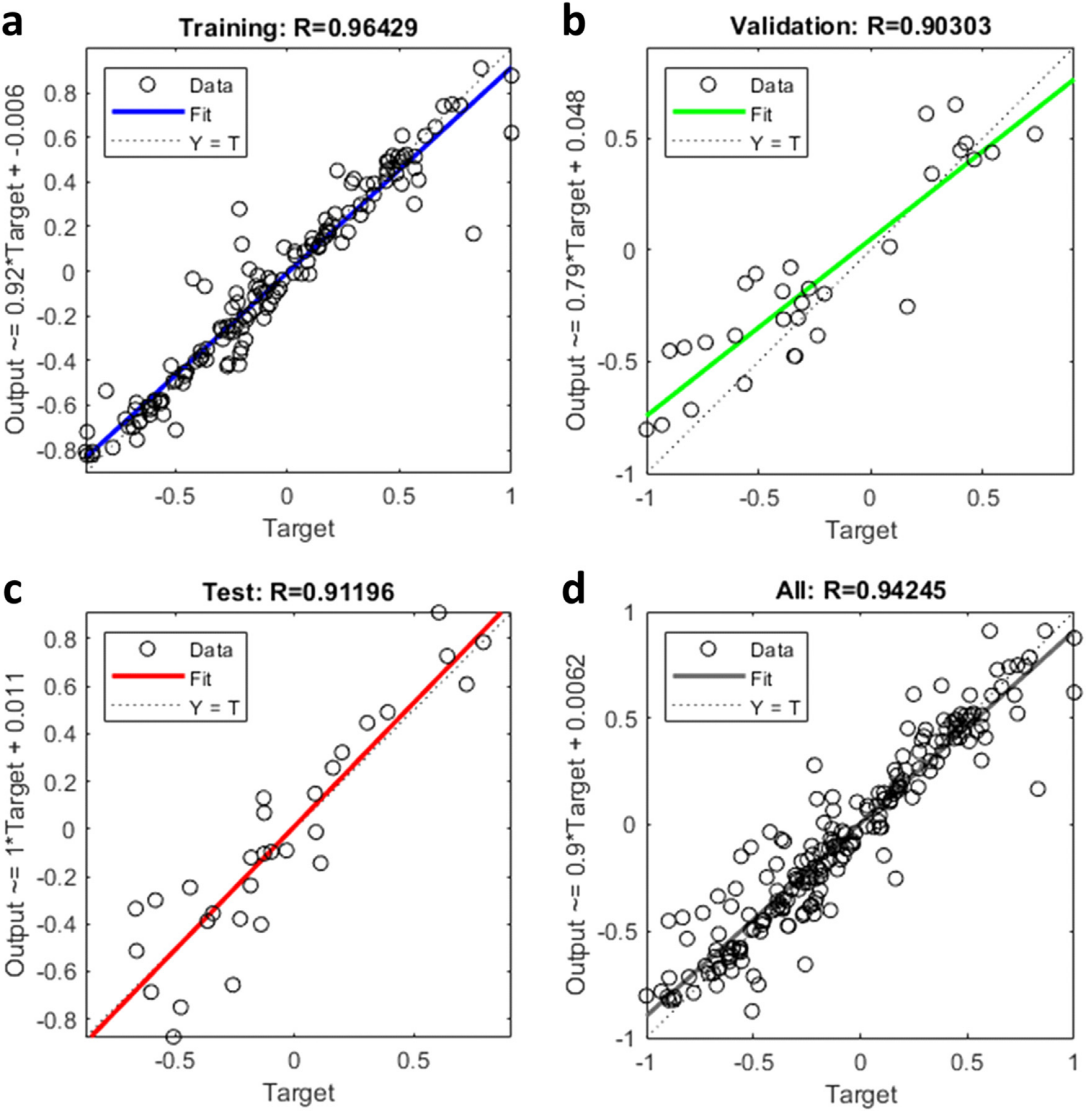
**BPNN model for the left-turn movement (N23)**. (a) Performance of the training. (b) Performance of the validation. (c) Performance of the test. (d) Performance of the all data.

The path dispersion models for the through and left-turn movements were developed. The performance results of the BPNN are presented in Table 4. All of them show a very high *R*^2^ for the training dataset with values of approximately 0.9. For the through movement, the best-performing BPNN model is N13 (*R*^2^ = 0.960, MAE = 0.035). It performs best in the training, validation, and testing datasets; therefore, this model is more applicable for describing the path dispersion at various intersections. Fig. 6 graphically shows the performance of the N13 model. For the left turn, the best-performing BPNN model is N23 (*R*^2^ = 0.942, MAE = 0.052). It performs best in the training, validation, and testing datasets. Besides, the *R*^2^ of all BPNN models is much higher than that of baseline fitting results, and the MAE of through movement and left-turn is only 10% and 8.07% of baseline method, which indicates that the BPNN model can explain the relationship between influencing factors and path dispersion more accurately. Fig. 7 graphically illustrates the performance of the N23 model. Based on the above results, N13 and N23 were selected as the path dispersion models for the through and left turn, respectively.

**Table 4:** **Performance results of the BPNN model and multiple linear regression**.

Movements	BPNN model architecture (input neurons *hidden neurons*output neurons)	Training R^2^	Validation R^2^	Testing R^2^	All Data			
					R^2^	MAE	R^2^ (MR)	MAE (MR)
Path dispersion of through movement	N11 (11×5*1)	0.930	0.797	0.857	0.898	0.054	0.282	0.35
	N12 (11×9*1)	0.982	0.883	0.726	0.923	0.041		
	N13 (11×13×1)	0.983	0.899	0.943	0.960	0.035		
Path dispersion of left-turn	N21 (10×5*1)	0.909	0.601	0.749	0.854	0.107	0.114	0.619
	N22 (10×9*1)	0.915	0.890	0.787	0.881	0.050		
	N23 (10×13×1)	0.964	0.903	0.912	0.942	0.052

**Fig. 8:**
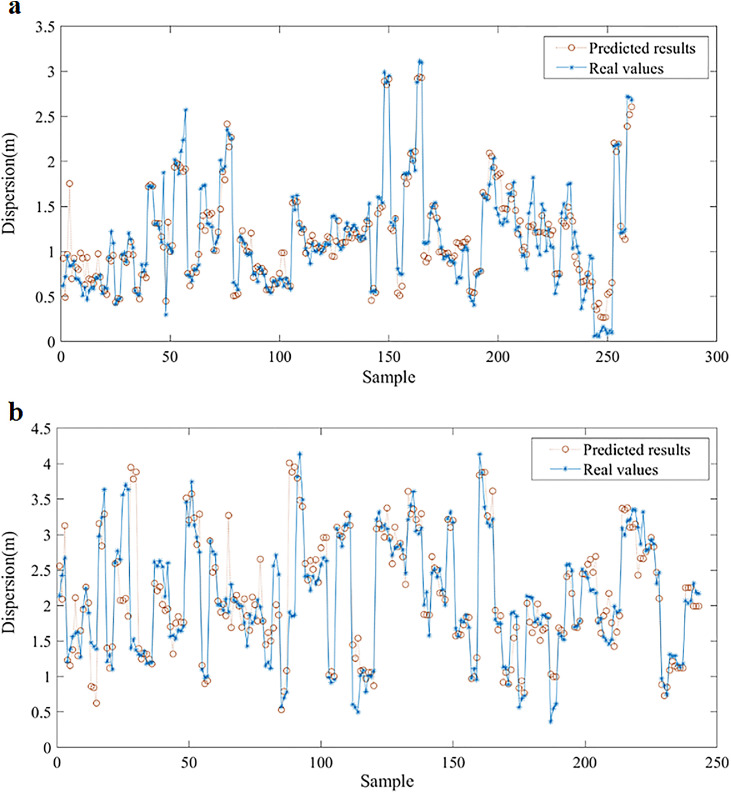
**Comparison between the predicted and real path dispersion**. (a) Through. (b) Left-turn.

**Fig. 9:**
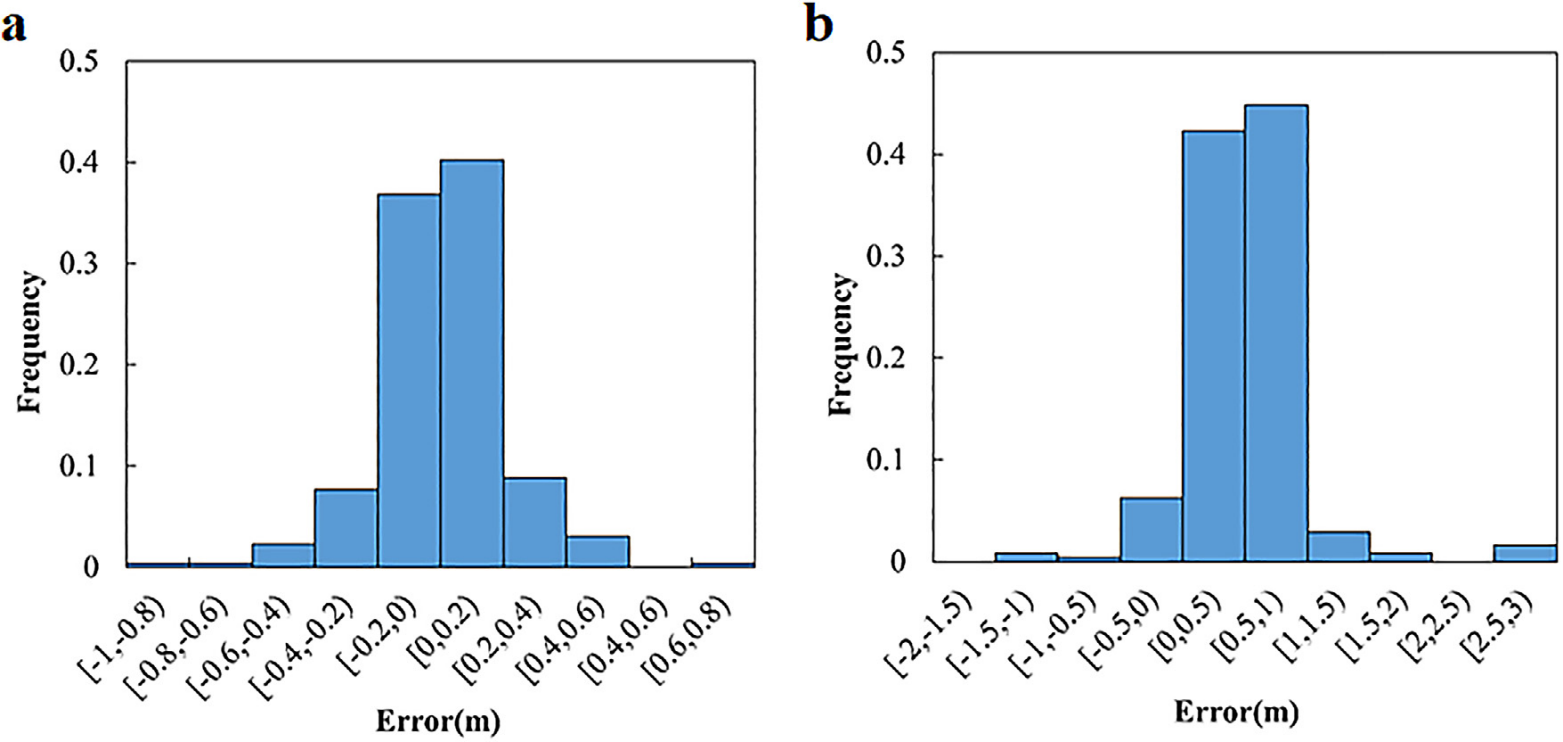
**Error frequency of the proposed models**. (a) Through. (b) Left-turn.

As shown in Fig. 8, it displays a comparison between the predicted and real path dispersion values ($\sigma_{m}^{p}$) of all video snippets. The average absolute errors of the path dispersion models for the through and left turn are 0.133 m and 0.290 m, respectively. Fig. 9 depicts the error frequencies of the two models. The absolute error of the path dispersion model for the through movement is concentrated between −0.2 and 0.2 m, whereas that for the left-turn is concentrated between 0 and 1 m. The prediction mean absolute percentage errors of the path dispersion models for the through and left turn are 14.67% and 17.65%, respectively.

## 4 Model explainability

To clarify the black box of the proposed BPNN model, this section analyzes the influence of each aspect of signalized intersections on the path dispersion based on weight visualization, the relative importance of input variables, and sensitivity analysis. Because the weights of neurons in the ANN are equivalent to the parameter coefficients in the regression model to some extent, the following methods in this section rely on the optimal BPNN models in Section 3.3 and the neuron weights in each layer of the model.

### 4.1 Neural interpretation diagram of the BPNN model

**Fig. 10:**
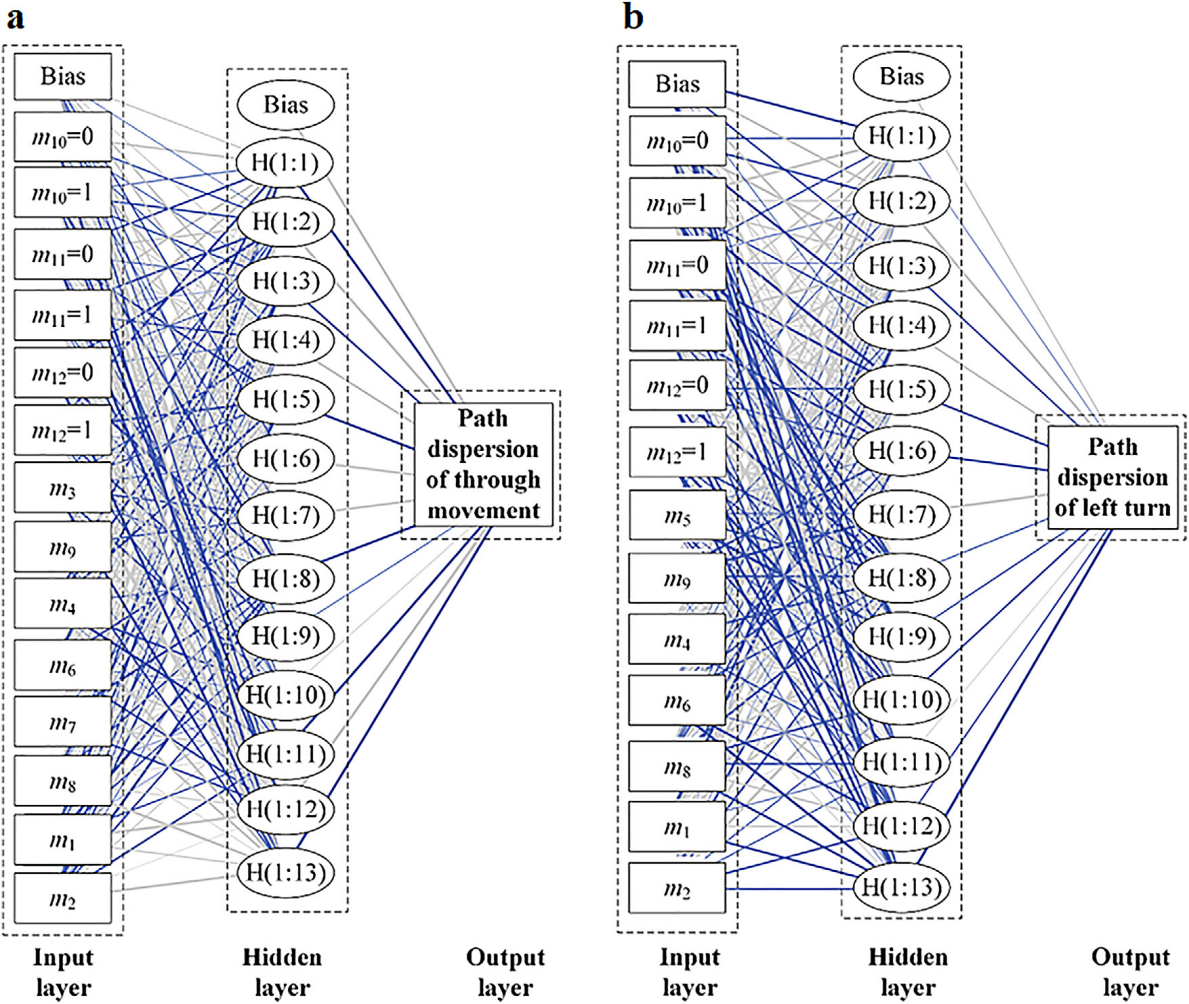
**Visual analysis of the BPNN model and its connection weights**. (a) Through. (b) Left-turn.

To visually interpret the optimized weights of the BPNN model between all input, hidden, and output neurons, the neural interpretation diagram [65,66] was applied to show the importance of the weights of neurons connected to each layer in the network, as illustrated in Fig. 10. The blue and gray lines represent positive and negative weights, respectively, whereas the thickness of the line reflects the magnitude of the weight.

For the through movement (Fig. 10a), the connecting lines between the following factors and the neurons in the hidden layer are denser and thicker: number of exit lanes (*m*_2_), offset degree between the approach and exit lanes (*m*_3_), traveled distance (*m*_4_), degree of saturation of the lane itself (*m*_6_), and setting of guideline (*m*_10_). This indicates that these factors have the greatest influence on the path dispersion of the through movement. Moreover, the first four factors are positively correlated with the dispersion of through paths because there are more blue lines between neurons in the hidden layer and the first three factors. The path dispersion of the through movement increases with an increase in the number of exit lanes, offset degree, traveled distance, and degree of saturation of the lane itself. In contrast, the last factor is negatively correlated with the dispersion of through paths because there are more gray solid lines between the neurons in the hidden layer and the last factor. The path dispersion of the through movement is greater when there is no guideline setting.

For the left-turn movement (Fig. 10b), the connecting lines between the following factors and the neurons in the hidden layer are denser and thicker: the number of exit lanes (*m*_2_), traveled distance (*m*_4_), left-turn angle (*m*_5_), conflicting flow volume (*m*_9_), and setting of guideline (*m*_10_). This suggests that these factors have a greater impact on the dispersion of left-turn paths. The first three factors are positively correlated with the dispersion of left-turn paths, whereas the last factor is negatively correlated.

Comparing Fig. 10a and b, the gray solid lines are denser at the guideline for the left turn than the through movement. Therefore, the influence of the guideline on the path dispersion of the left turn is greater than that of the through movement. Moreover, in contrast to the through movement, the blue connecting lines are denser and more concentrated between the conflicting flow volume and the neurons in the hidden layer of the path dispersion model for the left turn. This indicates that the through movement volume has a significant effect on the dispersion of the left-turn paths. They are positively correlated, that is, the more conflicting the flow volume, the greater the dispersion of the left-turn paths.

### 4.2 Relative importance of input variables

The neural interpretation diagram can help in qualitatively interpreting the relationship between each influencing factor and the dispersion of vehicular trajectories; however, it is not capable of quantitatively interpreting the neural network. This section presents the calculation of the relative importance of the output variable attributable to the given input variables using Garson's algorithm [67]:

(5) $$Q_{mk}=\frac{\sum_{j=1}^{S}\left(w_{mj}v_{jk}/\sum_{r=1}^{p}w_{rj}v_{jk}\right)}{\sum_{r=1}^{p}\sum_{j=1}^{S}\left(w_{mj}v_{jk}/\sum_{r=1}^{p}w_{rj}v_{jk}\right)}$$

where *Q_mk_* represents the importance of an input variable relative to the rest of the input variables, p is the number of neurons in the input layer, S is the number of neurons in the hidden layer, *w_mj_* is the connection weight between the input layer neuron *m* and hidden layer neuron *j*, and *v_jk_* is the connection weight between the hidden layer neuron *j* and output layer neuron *k*.

However, owing to different positive and negative values of the connection weights, the *Q_mk_* value calculated using Eq. (5) may become negative and cannot reflect the importance of the input variable *m_i_* to the output variable *y_k_*. To solve this problem, Goh [67] made the following improvements to:

(6) $$Q_{mk}=\frac{\sum_{j=1}^{S}\left(\left|w_{mj}v_{jk}\right|/\sum_{r=1}^{p}\left|w_{rj}v_{jk}\right|\right)}{\sum_{r=1}^{p}\sum_{j=1}^{S}\left(\left|w_{mj}v_{jk}\right|/\sum_{r=1}^{p}\left|w_{rj}v_{jk}\right|\right)}$$

**Fig. 11:**
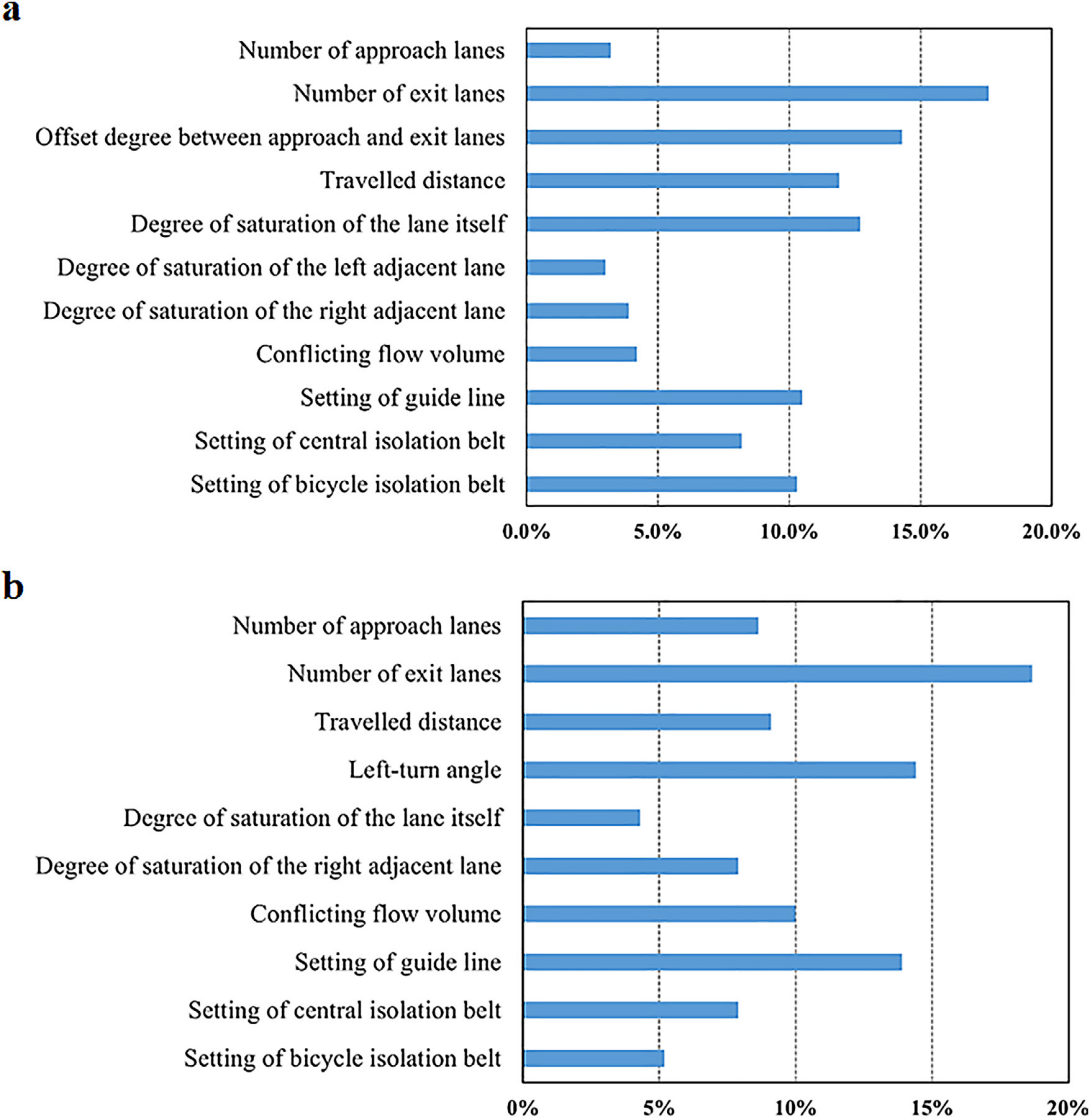
**Relative importance of influencing factors**. (a) Through. (b) Left-turn.

The calculation results are exhibited in Fig. 11. In accordance with the analysis result of the neural interpretation diagram, the relative importance analysis also shows that the number of exit lanes, traveled distance, and guideline are the three common key influencing factors (more than 40%) for the through and left-turn movements. The number of exit lanes has the greatest impact, with relative importance values of 17.6% and 18.7% for the path dispersion of the through and left-turn movements, respectively, is the greatest.

As shown in Fig. 11a, in addition to the common key influencing factors (the number of exit lanes, traveled distance, and guideline), the offset degree between the approach and exit lanes, saturation of the lane itself, and the bicycle isolation belt also have significant effects (more than 10%) on the dispersion of through paths. Interestingly, the relative importance of the bicycle isolation belt is 10.3%. This means that when a vehicle passes through an intersection, it is significantly affected by bicycles. The setting of the bicycle isolation belt can substantially reduce the dispersion of through paths.

As indicated in Fig. 11b, in addition to the common key influencing factors, the left-turn angle and conflicting flow volume have considerable impacts (more than 10%) on the dispersion of left-turn paths. Interestingly, the two factors, i.e., saturation of the lane itself and conflicting flow volume, have different impacts on the path dispersion for the through and left-turn movements. For the through movement, the relative importance of the saturation of the lane itself is 12.7%, ranking third in the relative importance of influencing factors. However, this factor ranks last for the left turn. The relative importance of the conflicting flow volume to the dispersion of left-turn paths ranks fourth, whereas it has little effect on the dispersion of through paths. This is because the left-turn movement should give way to the through movement. It makes sense that the dispersion of through paths is significantly affected by its traffic conditions, whereas that of the left turn is greatly influenced by its conflicting flow.

### 4.3 Sensitivity analysis

To elaborate on how the path dispersion changes with the influencing factors, this section provides a sensitivity analysis of the relationship between the output and input variables. When examining a certain input variable, the variable changes according to the measured range, whereas the other variables remain constant using their average values.

#### 4.3.1 Sensitivity analysis of dispersion for the through movement

**Fig. 12:**
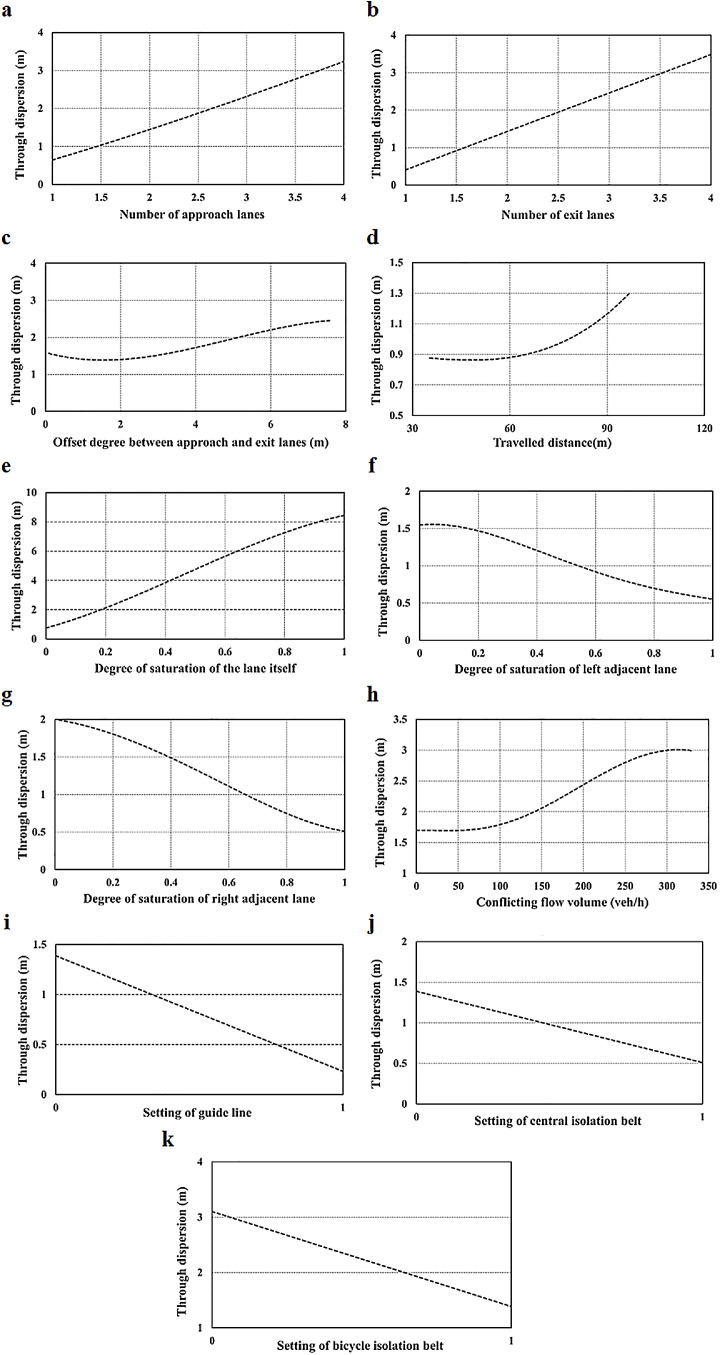
**Sensitivity analysis of path dispersion for through movement.** (a) Number of approach lanes. (b) Number of exit lanes. (c) Offset degree between approach and exit lanes. (d) Traveled distance. (e) Degree of saturation of the lane itself. (f) Degree of saturation of left adjacent lane. (g) Degree of saturation of right adjacent lane. (h) Conflicting flow volume. (i) Setting of guide line. (j) Setting of central isolation belt. (k) Setting of bicycle isolation belt.

Fig. 12 depicts the sensitivity analysis of the factors influencing the path dispersion of the through movement. It can be observed that the following factors are positively related to the through path dispersion: number of approach lanes, number of exit lanes, offset degree between the approach and exit lanes, traveled distance, degree of saturation of the lane itself, and conflicting flow volume. The degree of saturation of adjacent lanes, setting of guideline, central isolation belt, and bicycle isolation belt are negatively related to the through path dispersion.

For the geometric conditions, as exhibited in Fig. 12a–d, the through path dispersion increases with an increase in the number of entrance and exit lanes, offset degree between the approach and exit lanes, and traveled distance. Because vehicles have more choices to exit lanes with an increase in the number of approach and exit lanes, there is a higher probability for drivers to choose different target exit lanes. When the traveled distance increases, the uncertainty of the paths increases, which indicates the possibility of lane changing. This leads to an increase in the path dispersion.

For the traffic conditions, as illustrated in Fig. 12e–g, the through path dispersion increases with an increase in the degree of saturation of the lane itself, but it decreases with the degree of saturation of the left and right adjacent lanes. This is because as the traffic flow increases, its uncertainty also increases, subsequently increasing the path dispersion. In contrast, when the traffic flow of adjacent lanes increases, the chances of lane changing to adjacent lanes are considerably reduced. As a result, most through vehicles adopt the car-following behavior.

For the signalization condition, as displayed in Fig. 12h, the through path dispersion slightly increases with an increase in the conflicting flow volume. This is because the through movement has a higher crossing priority. Although the existence of left-turn vehicles has a negative effect on the smooth running of the through movement, the impact is only slight.

For the management conditions, as shown in Fig. 12i–k, when there are guidelines, central isolation belts, and bicycle isolation belts in the intersection, the dispersion of through paths is smaller. This shows that these intersection management and guidance facilities can help to make the traffic flow through movement run in order. Moreover, the bicycle isolation belt has a greater impact on the through path dispersion.

#### 4.3.2 Sensitivity analysis of path dispersion for the left-turn

**Fig. 13:**
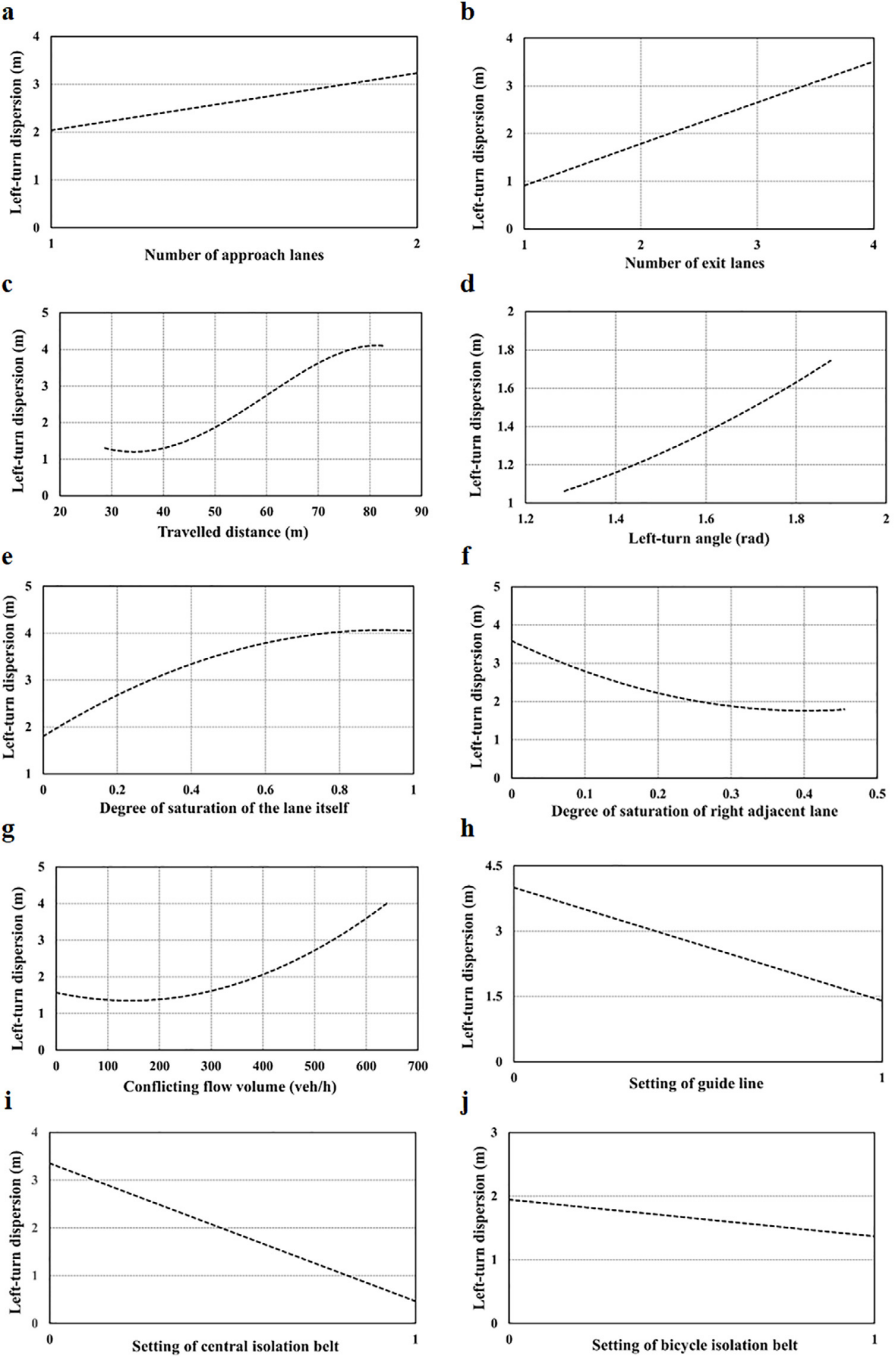
**Sensitivity analysis of path dispersion for the left turn.** (a) Number of approach lanes. (b) Number of exit lanes. (c) Traveled distance. (d) Left-turn angle. (e) Degree of saturation of the lane itself. (f) Degree of saturation of right adjacent lane. (g) Conflicting flow volume. (h) Setting of guide line. (i) Setting of central isolation belt. (j) Setting of bicycle isolation belt.

As shown in Fig. 13, it depicts the sensitivity analysis of the factors influencing the path dispersion of the left turn. It can be observed that the following factors are positively related to the path dispersion: number of approach and exit lanes, traveled distance, left-turn angle, degree of saturation of the lane itself, and conflicting flow volume. The degree of saturation of adjacent lanes, setting of guideline, central isolation belt, and bicycle isolation belt are negatively related to the path dispersion of the left turn.

For the geometric conditions, as presented in Fig. 13a–d the change tendencies of the left-turn path dispersion against the number of approach and exit lanes and traveled distance are the same as those of the through movement. In Fig. 13c, the dispersion of left-turn paths tends to be stable when the traveled distance reaches 80 m. The longer the traveled distance, the higher the uncertainty of the path. However, different from through vehicles, left-turn vehicles are also affected by the turning angle of the intersection. The possibility of temporarily changing the target exit lane is relatively small, thus the impact tends to stabilize. In Fig. 13d, it can be seen that the dispersion of left-turn paths increases with an increase in the left-turn radius.

For the traffic conditions, as indicated in Fig. 13e–f, the overall change tendencies of the degree of saturation of the lane itself (positive) and the degree of saturation of adjacent lanes (negative) are the same as those of the through movement. The left-turn path dispersion shows a trend of stabilizing as the two factors increase. It may be because the possibility of changing lanes is significantly reduced when the degree of saturation of adjacent lanes is high.

For the signalization condition, as illustrated in Fig. 13g, the dispersion of left-turn paths continues to increase with an increase in the conflicting flow volume. This is different from the dispersion of through paths, which tends to stabilize with an increase in conflicting flow. Moreover, the effect is more significant. This is because an increasing number of left-turn vehicles need to yield to through vehicles when the conflicting flow volume increases. Some vehicles prefer to keep the original left-turn path, some prefer to stay in the center of the intersection, whereas others prefer to slow down and make a detour. These heterogeneous driving behaviors cause path dispersion.

For the management conditions, as exhibited in Fig. 13h–j, these factors reduce the path dispersion of the left turn. However, their effects on the left turn are different from those on the through movement. For the through movement, the effect of the bicycle isolation belt is significant (2.7 times), but it is only slight on the path dispersion of the left turn (0.6 times). In contrast, the central isolation belt has a relatively greater impact on the dispersion of left-turn paths, but it has a smaller impact on the dispersion of the through paths. This is because left-turn vehicles generally tend to choose lanes in the middle of the road (near the central isolation belt), thus the central isolation belt has a stronger restriction on the left-turn trajectories than the bicycle isolation belt.

## 5 Conclusion

This paper put forward a BPNN model to describe the overview path dispersion of the through and left-turn movements based on real trajectory data collected from 20 intersections. Twelve influencing factors from four aspects, namely, geometric design of intersections, traffic operation, signal control, and traffic management, were considered. The accuracies of the path dispersion models for the through and left-turn movements were 85.33% and 82.35%, respectively. To determine the relationship between the influencing factors and path dispersion in the BPNN model, the model was investigated using the neural interpretation diagram, relative importance of influencing factors, and sensitivity analysis. The main findings are as follows:

(1) For through vehicles, the number of exit lanes, offset degree between the approach and exit lanes, and degree of saturation of the lane itself has the most significant positive effects on increasing the path dispersion of the through movement. Their relative importance values are 17.6%, 14.3%, and 12.7%, respectively.

(2) For left-turn vehicles, the number of exit lanes, left-turn angle, and setting of guideline have the greatest positive influence on increasing the path dispersion of the left-turn. Their relative importance values are 18.7%, 14.4%, and 13.9%, respectively.

(3) The degree of saturation of adjacent lanes, setting of guideline, central isolation belt, and bicycle isolation belt have a positive impact on the reduction in path dispersion. Management measures at intersections can help regulate vehicular paths. The bicycle isolation belt has a high impact on the path dispersion of the through movement, whereas the central isolation belt has a great impact on the path dispersion of the left turn.

This study analyzes the potential relationships between path dispersion and various traffic factors. However, in practice, there are additional factors, such as the weather, lighting conditions, and regional features. In future research, these additional factors should be considered. Moreover, based on this study, the driving behavior at intersections can be analyzed further and reasonable measures to prevent accidents and improve operational efficiency can be proposed.

## Declaration of competing interest

The authors declare that they have no conflicts of interest in this work.

## Appendix Supplementary materials

Image, application 1

## Footnotes

Supplementary material associated with this article can be found, in the online version, at doi:10.1016/j.fmre.2023.08.008.
