# Supplementary material for: Modeling and analysis of vehicle path dispersion at signalized intersections using explainable backpropagation neural networks
Source: Fundam Res. 2023 Nov 5;5(4):1645–58. doi: 10.1016/j.fmre.2023.08.008 (PMC12327834; doi:10.1016/j.fmre.2023.08.008)
Supplement: Supplementary file 1 [file mmc1.docx]

Supplementary materials

The detailed information of the surveyed intersections is presented in Table S1.

**Table S1: Detailed information of the surveyed intersections**

| No. | Intersection | Arm | NAL | NEL | DO | LP | GL | CID | BIB |
| --- | --- | --- | --- | --- | --- | --- | --- | --- | --- |
| 1 | Changyi Road-Fushan Road | East | 2 | 2 | 0 | 0 | 0 | 0 | 0 |
| 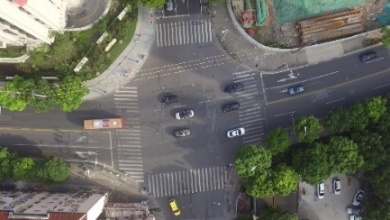 | | West | 2 | 2 | 0 | 0 | 0 | 0 | 0 |
|  |  | South | 2 | 1 | 0 | 0 | 0 | 0 | 0 |
|  |  | North | 2 | 1 | 0 | 0 | 0 | 0 | 0 |
| 2 | Taolin Road-Lingshan Road | East | 2 | 1 | 0 | 0 | 0 | 0 | 0 |
| 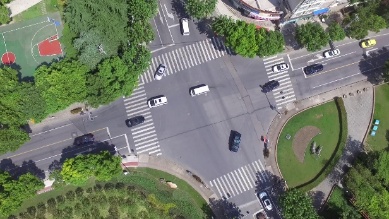 | | West | 2 | 1 | 0 | 0 | 0 | 0 | 0 |
|  |  | South | 2 | 1 | 0 | 0 | 0 | 0 | 0 |
|  |  | North | 2 | 1 | 0 | 0 | 0 | 0 | 0 |
| 3 | Guoxiao Road-Zhencheng Road | East | 3 | 0 | 0 | 0 | 0 | 0 | 0 |
|  | | West | 2 | 1 | N/A | 0 | 0 | 0 | 0 |
|  |  | South | 2 | 1 | 1 | 0 | 0 | 0 | 0 |
|  |  | North | 0 | 3 | N/A | N/A | 0 | 0 | 0 |
| 4 | Mouping Road-Changdao Road | East | 1 | 1 | 0 | 0 | 0 | 0 | 0 |
| 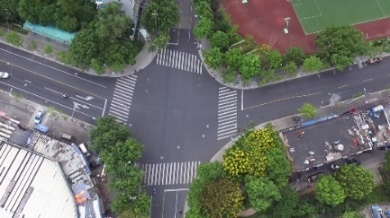 | | West | 1 | 1 | 0 | 0 | 0 | 0 | 0 |
|  |  | South | 2 | 1 | 1 | 0 | 0 | 0 | 0 |
|  |  | North | 2 | 2 | 1 | 0 | 0 | 0 | 0 |
| 5 | Yangtai Road-Zhentai Road | East | 3 | 1 | 0 | 0 | 0 | 0 | 0 |
| 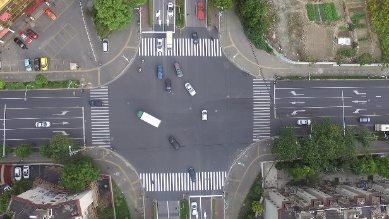 | | West | 3 | 1 | 0 | 0 | 0 | 0 | 0 |
|  |  | South | 2 | 2 | 0 | 0 | 0 | 1 | 1 |
|  |  | North | 2 | 2 | 0 | 0 | 0 | 1 | 1 |
| 6 | Yuanshen Road-Changyi Road | East | 2 | 2 | 0 | 0 | 1 | 0 | 0 |
| 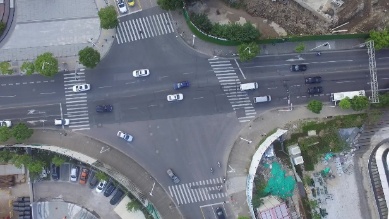 | | West | 2 | 2 | 0 | 0 | 1 | 0 | 0 |
|  |  | South | 2 | 2 | 0 | 0 | 0 | 0 | 0 |
|  |  | North | 2 | 1 | 0 | 0 | 0 | 0 | 0 |
|  | |  |  |  |  |  |  |  |  |
| 7 | Yinchun Road-Fangdian Road | East | 2 | 1 | 0 | 0 | 0 | 0 | 0 |
| 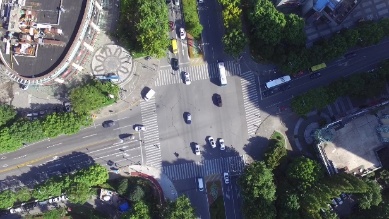 | | West | 3 | 2 | 0 | 0 | 0 | 0 | 0 |
|  |  | South | 2 | 2 | 0 | 0 | 0 | 1 | 1 |
|  |  | North | 2 | 2 | 0 | 0 | 0 | 1 | 1 |
| 8 | Zuchongzhi Road-Gaosi Road | East | 3 | 2 | 0 | 1 | 1 | 1 | 1 |
| 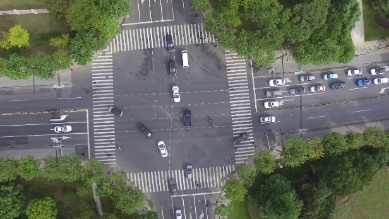 | | West | 3 | 2 | 0 | 1 | 1 | 1 | 1 |
|  |  | South | 3 | 1 | 1 | 0 | 1 | 0 | 0 |
|  |  | North | 3 | 2 | 1 | 0 | 1 | 0 | 0 |
| 9 | Shiboguan Road-Xueyeer Road | East | 3 | 2 | 0 | 0 | 0 | 1 | 0 |
|  | | West | 3 | 2 | 0 | 0 | 0 | 1 | 0 |
|  |  | South | 2 | 1 | 1 | 0 | 1 | 0 | 0 |
|  |  | North | 1 | 1 | 1 | 0 | 1 | 0 | 0 |
| 10 | Yinchun Road-Hehuan Road | East | 2 | 2 | 1 | 0 | 0 | 0 | 1 |
| 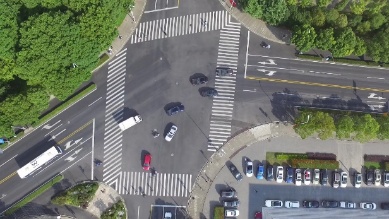 | | West | 2 | 2 | 1 | 0 | 0 | 0 | 1 |
|  |  | South | 2 | 1 | 0 | 0 | 0 | 0 | 0 |
|  |  | North | 2 | 1 | 1 | 0 | 0 | 0 | 0 |
| 11 | Zhongyuan Road-Nengjiang Road | East | 3 | 2 | 0 | 0 | 0 | 0 | 1 |
| 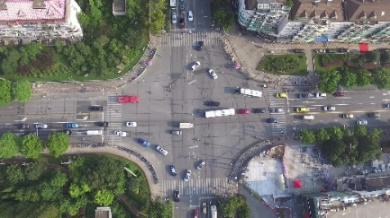 | | West | 3 | 2 | 0 | 0 | 0 | 1 | 1 |
|  |  | South | 3 | 2 | 0 | 1 | 0 | 1 | 1 |
|  |  | North | 3 | 2 | 0 | 1 | 0 | 1 | 1 |
| 12 | Youyi Road-Tieli Road | East | 3 | 2 | 0 | 1 | 0 | 1 | 1 |
| 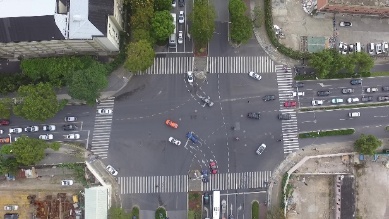 | | West | 5 | 2 | 0 | 1 | 1 | 1 | 1 |
|  |  | South | 3 | 2 | 0 | 0 | 1 | 1 | 1 |
|  |  | North | 2 | 2 | 1 | 0 | 1 | 0 | 0 |
| 13 | Nanyangjing Road-Yushan Road | East | 2 | 1 | 0 | 0 | 0 | 0 | 0 |
| 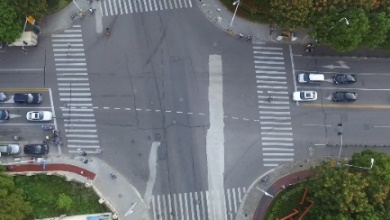 | | West | 2 | 2 | 0 | 0 | 0 | 0 | 0 |
|  |  | South | 3 | 2 | 0 | 0 | 0 | 0 | 1 |
|  |  | North | 2 | 1 | 0 | 1 | 1 | 0 | 0 |
| 14 | Mudanjiang Road-Baoyang Road | East | 3 | 2 | 0 | 0 | 0 | 1 | 1 |
| 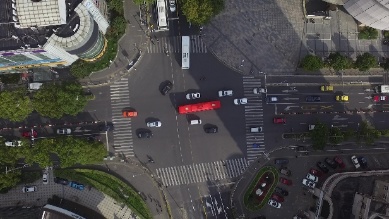 | | West | 2 | 2 | 0 | 0 | 0 | 1 | 1 |
|  |  | South | 2 | 2 | 1 | 0 | 0 | 0 | 0 |
|  |  | North | 2 | 1 | 0 | 0 | 1 | 0 | 0 |
| 15 | Gaokezhong Road-Zhangdong Road | East | 4 | 3 | 0 | 1 | 1 | 1 | 1 |
| 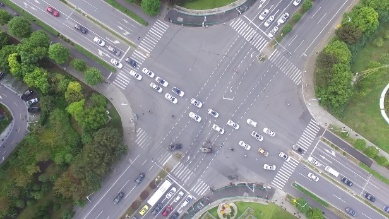 | | West | 4 | 3 | 0 | 1 | 1 | 1 | 1 |
|  |  | South | 3 | 2 | 0 | 0 | 0 | 1 | 1 |
|  |  | North | 3 | 2 | 0 | 0 | 0 | 1 | 1 |
| 16 | Xinjinqiao Road-Shenjiang Road | East | 3 | 2 | 1 | 1 | 0 | 0 | 1 |
| 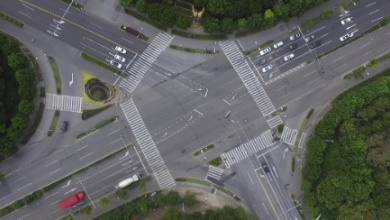 | | West | 3 | 2 | 1 | 1 | 0 | 0 | 1 |
|  |  | South | 4 | 3 | 0 | 1 | 0 | 1 | 1 |
|  |  | North | 4 | 3 | 0 | 1 | 0 | 1 | 1 |
| 17 | Jingxiu Road-Fangdian Road | East | 4 | 3 | 0 | 1 | 0 | 1 | 0 |
| 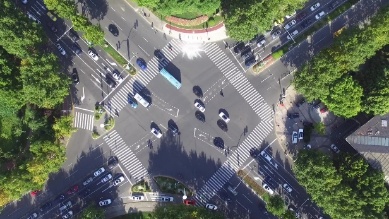 | | West | 4 | 3 | 0 | 1 | 0 | 1 | 0 |
|  |  | South | 3 | 2 | 0 | 1 | 0 | 0 | 0 |
|  |  | North | 3 | 2 | 0 | 1 | 0 | 1 | 1 |
| 18 | Zhangyang Road-Gushan Road | East | 5 | 4 | 0 | 1 | 0 | 1 | 0 |
| 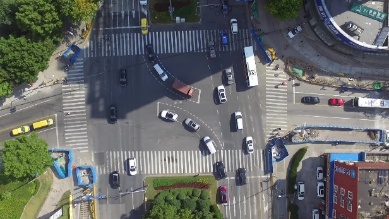 | | West | 5 | 4 | 0 | 1 | 1 | 1 | 0 |
|  |  | South | 1 | 1 | 1 | 0 | 0 | 0 | 0 |
|  |  | North | 1 | 1 | 1 | 0 | 0 | 0 | 0 |
| 19 | Gaokezhong Road-Jinke Road | East | 3 | 2 | 0 | 1 | 0 | 1 | 1 |
| 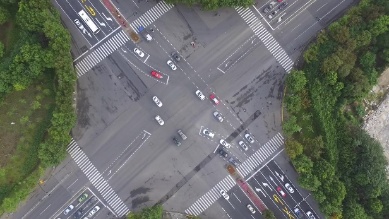 | | West | 3 | 2 | 0 | 1 | 0 | 1 | 1 |
|  |  | South | 4 | 3 | 0 | 1 | 1 | 1 | 1 |
|  |  | North | 4 | 2 | 0 | 1 | 0 | 1 | 1 |
| 20 | Youyi Road-Tieshan Road | East | 4 | 2 | 1 | 1 | 1 | 1 | 1 |
| 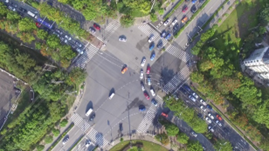 | | West | 3 | 2 | 1 | 1 | 0 | 1 | 1 |
|  |  | South | 3 | 2 | 0 | 0 | 1 | 1 | 1 |
|  |  | North | 3 | 2 | 0 | 0 | 0 | 1 | 1 |

Note: NAL and NEL represent the number of approach lanes and exit lanes, respectively. E, S, W, and N represent east, south, west, and north, respectively. The distance offset between the approach and exit lanes (DO) is defined as “1-large” when the offset exceeds one lane; otherwise “0-small”. LP, GL, CID, and BIB are binary variables to represent the presence of a protected left-turn phase, guideline, central isolation belt, and bicycle isolation belt, respectively. For example, if there exist bicycle isolation belts, BIB=1; otherwise, BIB=0.
